# A physical perspective to understand myelin II: The physical origin of myelin development

**DOI:** 10.3389/fnins.2022.951998

**Published:** 2022-10-03

**Authors:** Yonghong Liu, Wenji Yue, Shoujun Yu, Tian Zhou, Yapeng Zhang, Ran Zhu, Bing Song, Tianruo Guo, Fenglin Liu, Yubin Huang, Tianzhun Wu, Hao Wang

**Affiliations:** ^1^Institute of Biomedical and Health Engineering, Shenzhen Institutes of Advanced Technology (SIAT), Chinese Academy of Sciences (CAS), Shenzhen, China; ^2^Key Laboratory of Health Bioinformatics, Chinese Academy of Sciences, Shenzhen, China; ^3^Graduate School of Biomedical Engineering, University of New South Wales, Sydney, NSW, Australia

**Keywords:** myelin development, g-ratio, electrical stimulation, neural degenerative disorder, E-field

## Abstract

The physical principle of myelin development is obtained from our previous study by explaining Peter’s quadrant mystery: an externally applied negative and positive E-field can promote and inhibit the growth of the inner tongue of the myelin sheath, respectively. In this study, this principle is considered as a fundamental hypothesis, named Hypothesis-E, to explain more phenomena about myelin development systematically. Specifically, the g-ratio and the fate of the Schwann cell’s differentiation are explained in terms of the E-field. Moreover, an experiment is proposed to validate this theory.

## Introduction

**FIGURE 1:**
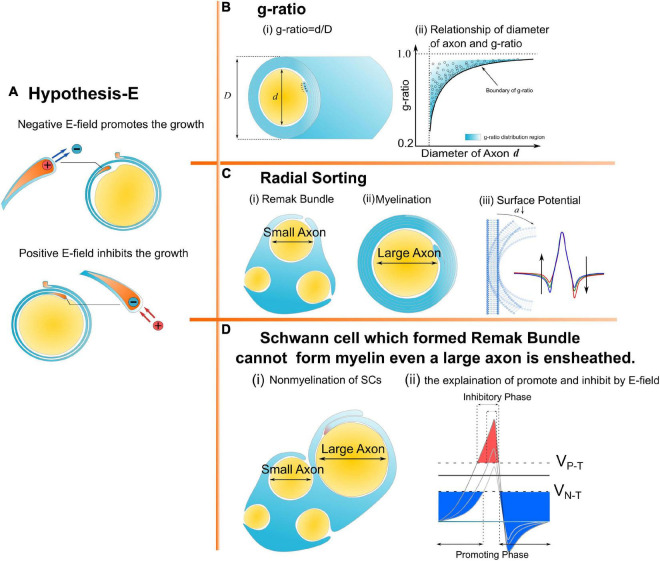
A series of morphological characteristics of myelin explained a mathematical and physical perspective. **(A)** Hypothesis-E: The effect of E-field on myelin growth; **(B,C)** phenomena explained by Hypothesis-E: **(B)** g-ratio: The thickness of myelin sheath has a specific relationship with the diameter of the axon. **(C)** Radial Sorting: Myelin selectively myelinated axon based on axonal diameter; **(D)** SC of remak bundle cannot form myelin even when a large axon is ensheathed.

Myelin is an insulating sheath forming around axons. Its biological function in neural systems and the growing mechanism have attracted increasing attention in the field of neuroscience (Lemke, 1988; Colognato and Franklin, 2004; Dutta et al., 2018; Stadelmann et al., 2019; Fields and Bukalo, 2020; Liu et al., 2021a). Previous studies reported a series of experimental observations about the micro-structures of myelin. For example, (1) The spiraling directions of neighboring myelin sheaths has a certain pattern. That is, the neighboring myelin sheaths on the same axon have the opposite spiraling direction (Richards et al., 1983), while the neighboring myelin sheaths on the adjacent axons have the same spiraling directions (Uzman and Nogueira-Graf, 1957; Bunge et al., 1989; Armati and Mathey, 2013); (2) For oligodendrocytes (OLs), the inner and outer tongues tend to be located within the same radial quadrant (Peters, 1961, 1964; Hildebrand, 1971; Webster, 1971; Fraher, 1972); (3) The axons of varying calibers tend to have myelin sheaths of the same thickness, resulting in the g-ratio phenomenon (FitzGibbon and Nestorovski, 2013; Stikov et al., 2015; Andersson et al., 2020); (4) Only the axon with sufficient caliber can be myelinated, resulting in the radial sorting phenomenon (Feltri et al., 2016; Harty et al., 2019; Ommer et al., 2019); and (5) For Schwann cells (SCs), one SC can only myelinate one axon. If the SC forms the remak bundle, it can never form the myelination, even if a large axon is ensheathed (Harty et al., 2019). These experimental observations indicate a multifaceted mechanism underlying myelin growth. For example, (1) the non-random spiraling phenomenon suggests that myelin growth can be influenced by the interaction between spatially closed myelin sheaths. (2) The same quadrant phenomenon indicates that myelin growth can be influenced by the relationship between the inner and outer tongues. (3) The g-ratio phenomenon indicates a possible correlation between inner tongue growth and the number of myelin lamellae. (4) The radial sorting phenomenon indicates a possible correlation between the myelin growth and the curvature of axons. (5) The characteristic SC properties in myelination and the remak bundle indicate the effect of the surrounding environment in formulating the growth of the inner tongue. Previous studies about mechanisms underlying myelin growth mainly focused on studying contributions from different molecules or proteins (Höke et al., 2003; Colognato and Franklin, 2004; Zheng et al., 2008; Orita et al., 2013; Hines et al., 2015; Feltri et al., 2016; Harty and Monk, 2017; Harty et al., 2019) without providing reasonable explanations for these phenomena. All these phenomena indicate an action at a distance, which is a kind of physical field, is dominating the formation of myelin growth. Thus, a study from a physical perspective can provide a substantial body of new knowledge yet to be discovered. In our previous study, the non-random spiraling phenomenon and the same quadrant phenomenon were explained from the perspective of the electromagnetic field (Wang et al., 2021) and electric field (Liu et al., 2021a). The former reveals the function of cytoplasmic channels in myelin sheath as a coil inductor and the role of the magnetic field in the neural signal. The latter reveals that the electric field modulates the growth of myelin. In this study, we further extend the hypothesis in our previous work, named as Hypothesis-E, to conduct *in silico* investigations of the physical origins of the unexplained myelin observations mentioned above. We name it Hypothesis-E, “E” refers to “electric.” In Hypothesis-E, an external negative E-field promotes myelin growth, while an external positive E-field inhibits myelin growth (Figure 1A). This study proposed three new hypotheses based on Hypothesis-E to further explain the physical origins of a series of morphological characteristics (Figures 1B–D) of the myelin.

## Hypothesis-E_*N*_ to explain g-ratio

### g-ratio

The myelin g-ratio, defined as the ratio between the inner and the outer diameter of the myelin sheath, has been reported in many experimental studies (FitzGibbon and Nestorovski, 2013; Stikov et al., 2015; Andersson et al., 2020). This precise relationship between axonal diameter and myelin sheath thickness has been reported is one of the most enigmatic questions: how is the myelinating glial cell instructed to make precisely the correct number of wraps? Transplantation of oligodendrocytes into nerve tracts containing axons of different sizes demonstrates that the number of wraps is determined by the axon but not by the glial cell because the transplanted glial cells elaborate myelin sheaths appropriate for their new location (Fanarraga et al., 1998). A key axonal signal for regulating myelin sheath thickness, the growth factor neuregulin (Ngr1), is now identified by Zuckerkandl and Pauling (1965). However, the detailed mechanisms of controlling the myelin wrapping by the axonal signal remain unclear.

### Hypothesis-E_*N*_

**FIGURE 2:**
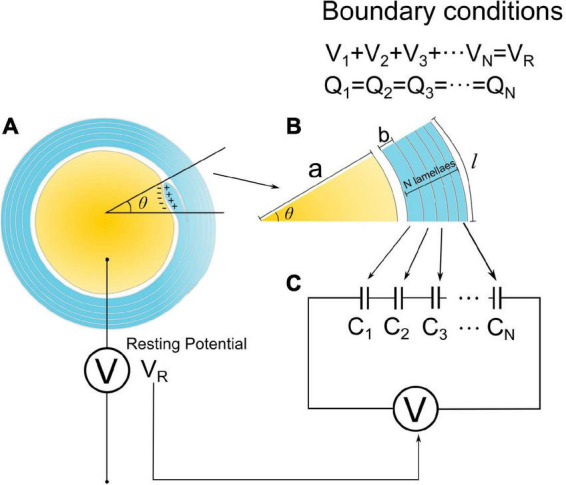
The model to explain g-ratio. **(A)** The cross-section of a myelinated axon in the static condition, the resting potential is equivalent to a voltage source; **(B)** a section of myelin cross-section with a radial angle of θ; **(C)** the equivalent circuit modeling the myelin cross-section.

The cross-section of a myelinated axon in the resting state (no action potential is activated) is shown in Figure 2A. The intracellular potential is more negative than the extracellular potential, resulting in a negative E-field on the inner tongue. This negative E-field is the driven force making the inner tongue grow and wrap around the axon to form myelination. Then the Hypothesis-E_*N*_ (“N” refers to “negative”) is described as follows:

*The inner tongue of myelin is driven by a negative E-field from the axon in the resting state. The strength of the E-field on the inner tongue is proportional to its growth rate. When the E-field is lower than a threshold, the growth of the inner tongue terminates.*

### Modeling the relationship between g-ratio and the E-field

Figure 2B shows a mature myelinated axon with the number of myelin lamellae as ***N***. The axonal radius is ***a***, and the thickness of a single myelin lamella is ***b***. Then the total myelin thickness, ***D***, is **b** × **N**. We assume that the axonal cross-section is centrally symmetric. So we only simulate the local axon with a radial angle as θ, as shown in Figure 2B. The capacitance, ***C***, of each layer is proportional to its area. Since the longitudinal length of each layer is identical, the capacitance of each layer is proportional to the arc length ***l***:


C∝A⁢r⁢e⁢a∝l

Then for the ***n**^th^* layer, the capacitance, ***C***_*n*_, is proportional to its arc length ***l***_*n*_:


$$C_{n}\propto l_{n}=\theta \times \left(a+\left(n-1\right)\,b\right)$$

The voltage, ***V***_*n*_, on the ***n**^th^* layer is $V_{n}=\frac{Q_{n}}{C_{n}}$.

Here ***Q***_*n*_ is the charge on the capacitor. So the voltage, ***V***_1_, on the first layer is $V_{1}=\frac{Q_{1}}{C_{1}}$.

Since all capacitors are connected in series, as shown in Figure 2C, the two boundary conditions are:

(1). The charge on each capacitor is the same, assigned with the value of ***Q***:

$$Q=Q_{1}=Q_{2}=Q_{3}=\cdots =Q_{N}$$

(2). The resting potential, ***V***_*R*_, is equivalent to a voltage source connected with these series-connected capacitors, as shown in Figure 2C, so ***V***_*R*_ is the sum of the voltage on all capacitors:

$$V_{R}=\sum_{n=1}^{N}V_{n}=\sum_{n=1}^{N}\frac{Q_{n}}{C_{n}}=Q\times \sum_{n=1}^{N}\frac{1}{C_{n}}$$

The charge, ***Q***, on each capacitor is:


$$Q=\frac{V_{R}}{\sum_{n=1}^{N}\frac{1}{C_{n}}}$$

The voltage, ***V***_1_, on the first layer, which is the inner tongue, is as shown below:


(1) $$V_{1}=\frac{Q}{C_{1}}=\frac{V_{R}}{C_{1}\times \sum_{n=1}^{N}\frac{1}{C_{n}}}=\frac{V_{R}}{a\times \sum_{n=1}^{N}\frac{1}{\left(a+\left(n-1\right)\,b\right)}}$$

when the voltage potential, ***V***_*R*_, and the thickness of a single myelin lamella, ***b*** are constants, the voltage on the inner tongue, ***V***_1_, is only a function of the number of layers ***N***, axonal radius ***a***, and monotonically decreases with the number of layers, ***N***. Here the threshold E-field proposed in Hypothesis-E_*N*_ is defined as ***V**_*N*–*T*_* (“N” refers to “negative” and “T” refers to “threshold”).

And the ratio between ***V**_N–*T*_* and ***V***_*R*_ is defined as η*_*N*–*T*_*:


(2) $$\eta_{N-T}=\frac{V_{N-T}}{V_{R}}$$

Then the criteria for the max number of myelin lamellae ***N***_*max*_ is:


(3) $$\left\{ \begin{array}{c} V_{1}\geq V_{N-T}\,w\,h\,e\,n\,N=N_{m\,a\,x}\\ V_{1}\leq V_{N-T}\,w\,h\,e\,n\,N=N_{m\,a\,x}+1 \end{array}\right.$$

Substitute (1) and (2) into (3) and get:


(4) $$\left\{ \begin{array}{c} \frac{1}{a\times \sum_{n=1}^{N_{m\,a\,x}}\frac{1}{\left(a+\left(n-1\right)\,b\right)}}\geq \eta_{N-T}\\ \frac{1}{a\times \sum_{n=1}^{N_{m\,a\,x}+1}\frac{1}{\left(a+\left(n-1\right)\,b\right)}}\leq \eta_{N-T} \end{array}\right.$$

(4) can be further simplified as follow:


(5) $$\frac{1}{a\times \sum_{n=1}^{N_{m\,a\,x}}\frac{1}{\left(a+\left(n-1\right)\,b\right)}}\approx \eta_{N-T}$$

As seen, the value ***N***_*max*_ is a function of ***a*** and η *_*N*–*T*_*, while ***b*** is constant:


$$N_{m\,a\,x}=f_{1}\,\left(a,\eta_{N-T}\right)$$

Then *g-ratio* is also a function of ***a*** and η *_*N*–*T*_*:


$$g_{r\,a\,t\,i\,o}=\frac{a}{a+D}=\frac{a}{a+b\times N_{m\,a\,x}}=\frac{a}{a+b\times f_{1}\,\left(a,\eta_{N-T}\right)}=f_{2}\,\left(a,\eta_{N-T}\right)$$

**FIGURE 3:**
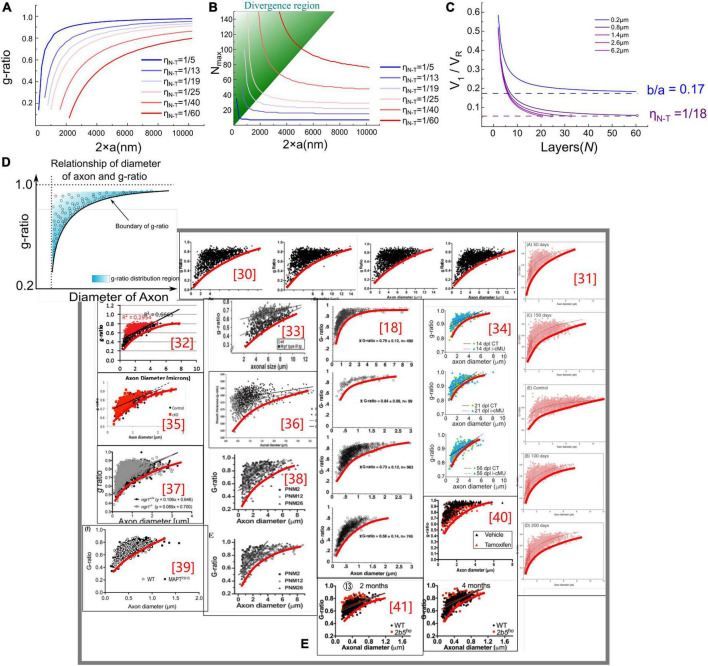
**(A,B)** g-ratio values and the maximum number of myelin lamellae, *N*_*max*_ values, given different η *_*N*–*T*_*. **(C)** The relationship between ***N***_*max*_ and ***V**_1_/V_*R*_.* When b/a < η *_*N*–*T*_*, ***N*_*max*_** is a finite number, otherwise ***N***_*max*_ is infinite. **(D)** Illustration of the claim: the measured statistical data of g-ratio shall locate above the g-ratio curve; **(E)** the measured statistical data of g-ratio in publications (Michailov et al., 2004; Paus and Toro, 2009; Ikeda and Oka, 2012; FitzGibbon and Nestorovski, 2013; Barbizan et al., 2014; Bercury et al., 2014; Nave and Werner, 2014; Ronchi et al., 2014; Xie et al., 2014; Lee et al., 2017; Dimas et al., 2019; Marro et al., 2019; Ferreira et al., 2021).

To enable calculating these two functions, we need to obtain the constant of ***b***. Based on previous studies, we set ***b*** = 17 nm as a typical value (Nave and Werner, 2014). The g-ratio and ***N***_*max*_ simulation is shown in Figures 3A,B.

### Results

In Figure 3A, the g-ratio curve monotonically increases with axonal radius ***a***. The curve of ***N***_*max*_ has a decreasing slope with ***a***, approaching a constant value determined by η ***_*N*–*T*_***. It is emphasized that ***N***_*max*_ is an axon’s maximum number of myelin lamellae. The actual measured number of myelin lamellae ***N*** should be no more than ***N*_*max*_** shown in Figure 3B, *N*≤*N*_*max*_. Thus, g-ratio curves shown in Figure 3A are a minimum value, which is a lower limit. As illustrated in Figure 3D, all measured data points of the g-ratio shall be higher than the g-ratio curve in Figure 3A. By fitting the curve of this lower edge, the actual η ***_*N*–*T*_*** can be obtained. In Figure 3E, we validate our simulations with the experimental data from previously published studies of myelinated axons (Sanders and Whitteridge, 1946; Zhao et al., 1995; Michailov et al., 2004; Chomiak and Hu, 2009; Paus and Toro, 2009; Ikeda and Oka, 2012; FitzGibbon and Nestorovski, 2013; Barbizan et al., 2014; Bercury et al., 2014; Ronchi et al., 2014; Xie et al., 2014; Lee et al., 2017; Klok et al., 2018; Dimas et al., 2019; Marro et al., 2019; Ferreira et al., 2021). We found a clear edge can be formed (the red fitting curves are plotted by ourselves for an indicator of boundary). As mentioned above, by fitting this lower edge, the threshold voltage, which is an important characteristic of the target nervous system, can be obtained. This characteristic is not recognized yet in conventional theories and models. Noticeably, ***N***_*max*_ goes infinite when ***a*** approaches zero, indicating that the axon with a very small diameter can have infinitely thick myelin. However, the axons with a radius within the divergence region in Figure 3B are unmyelinated. We will make a more detailed discussion in the next section.

### Discussion

#### Why does the divergence happen?

The condition to achieve ***N***_*max*_ is to meet the condition of Equation (5), as written again here:


(5) $$\frac{1}{a\times \sum_{n=1}^{N_{m\,a\,x}}\frac{1}{\left(a+\left(n-1\right)\,b\right)}}\approx \eta_{N-T}$$

However, the limit of η *_*N*–*T*_* when ***N***_*max*_ approaches infinite is as follows:


$$\lim_{N_{m\,a\,x}\to \infty }\eta_{N-T}=\underset{N_{m\,a\,x}\to }{\text{lim}}\frac{1}{a\times \sum_{n=1}^{N_{m\,a\,x}}\frac{1}{\left(a+\left(n-1\right)\,b\right)}}=\frac{b}{a}$$

where ***b/a*** is the lower limit of η *_*N*–*T*_*. If the actual η *_*N*–*T*_* is above this lower limit, ***V***_1_ can reach ***V***_N_*_–*T*_* when ***N***_*max*_ is a finite number; then, the myelin growth stops (Eq. 6). However, if the actual is η *_*N*–*T*_* lower than this lower limit, ***V***_1_ can never be reduced to ***V**_*N*–*T*_*, whatever ***N***_*max*_ is; the myelin growth never stops (Eq. 7).


(6) $$w\,h\,e\,n\,\frac{b}{a}<\eta_{N-T};N=f\,i\,n\,i\,t\,e\,n\,u\,m\,b\,e\,r$$

(7) $$w\,h\,e\,n\,\frac{b}{a}\geq \eta_{N-T};N\to \infty$$

where the occurrence of divergence is determined by the ratio between the thickness of single-layer myelin, ***b***, and the axonal radius, ***a***. When ***a*** is large enough to meet Eq. (6), the calculation of ***N***_*max*_ is convergent. Otherwise, the divergence happens when ***a*** is a small number, which is the case of unmyelinated axons. A more intuitive modeling result is shown in Figure 3C. Since ***V***_1_ decreases with the growth of myelin lamellae, the ratio of ***V**_1_/**V**_*R*_* will decrease with ***N***. Then this ratio reaches the value of η *_*N*–*T*_*, the curve stops at the value of ***N***_*max*_. As seen, the curve of the axonal diameter of 0.8, 1.4, 2.6, and 6.2 μm can have a finite value of ***N***_*max*_. However, when axon diameter is 0.2 μm, the curve of ***V**_1_/**V**_*R*_* approaches $\frac{b}{a}=0.17$ (***b*** = 17 nm and axonal radius ***a*** = 100 nm), which is higher than η *_*N*–*T*_* = 1/18≈0.056, the growth cannot be stopped.

#### The relation between the divergence region and unmyelinated axons

Since the modeling result can closely predict the biological observations of the g-ratio and myelin thickness at different axonal diameters, we tend to explore the biological meaning hidden behind the divergence region. It is observed that the number of myelin lamellae suddenly decreases to zero when the axonal diameter is lower than a threshold, indicating that some unknown factors dominate the growth of smaller myelin and forbid the process of myelination.

Interestingly, Hypothesis-E_*N*_ suggests that the axon of very small diameter can have infinitely thick myelin, which disagrees with biology. Therefore, some unknown factor that inhibits myelin growth during myelin development is introduced when the axonal diameter is lower than a certain value. We will discuss this unknown factor in section “A revision of the g-ratio model.”

#### An introspection of this model

The origin of the g-ratio is the myelin’s growth rate inversely proportional to its layers. That is, the promoting factor of myelin growth decays with its layers. Meanwhile, the inner tongue is the growing terminal of the myelin, indicating this promoting factor exerts its function on the inner tongue. In our model, the voltage, ***V***_1_, on the inner tongue meets these boundary conditions. Any alternative theories shall also meet the above-mentioned boundary conditions. Since this ***V***_1_ is obtained from Hypothesis-E_*N*_, so it is renamed as ***V***_*EN*_ in this article to avoid confusion.

## Hypothesis-E_*D*_ to explain radial sorting

### Radial sorting

Radial sorting is the process by which Schwann cells choose larger axons to myelinate during development (Monk et al., 2015). During this process, SCs proliferate and expand cellular extensions into bundles of unsorted axons to detach individual axons and establish the 1:1 relationship (one SC can only myelinated one axon) required for myelination (Webster et al., 1973). Axons with a diameter of < 1 μm remain in bundles, and SCs in contact with these axons differentiate into unmyelinated SCs, called remak bundles (Griffin and Thompson, 2008). This radial sorting process is reported to be tightly regulated and depends on signals from axons as well as the extracellular matrix (Ghidinelli et al., 2017).

### Hypothesis-E_*D*_

With the radial sorting process, SCs can recognize functionally identified axons just by their calibers. Axons of large caliber possess a promoting factor, while the axons of small caliber possess an inhibiting factor to the myelin growth. Based on Hypothesis-E, we can predict that larger axons can possess a more negative voltage than that of smaller axons. Then Hypothesis-E_*D*_ (“D” refers to “dipole”) refers to:

*When SCs get close to the surfaces of axons, axons of larger caliber will exert a special “E-field,” which is more negative than that of the axons of smaller caliber, to the cell membrane of SCs. Thus, SCs tend to grow and wrap on larger axons. When the caliber of axons is lower than a threshold, the amplitude of the negative E-filed is too low to enable the growth of SCs on their surfaces.*

### Modeling the relationships between the radial sorting and the dipole potential

**FIGURE 4:**
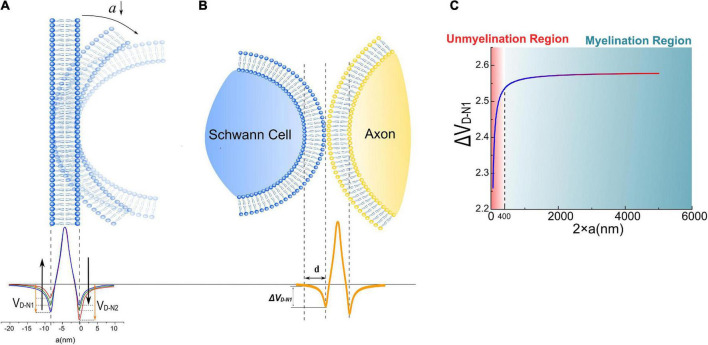
**(A)** The dipole potential generated by the bending of the cell membrane; **(B)** when a Schwann cell contact with the cell membrane of an axon, a portion of the surface potential, *V_*D*–*N1*_*, will be exerted upon Schwann cell’s membrane, labeled as Δ ***V**_*D*–*N1*_*; **(C)** the simulation result of Δ ***V**_*D*–*N1*_* is a function of axonal diameters.

**FIGURE 5:**
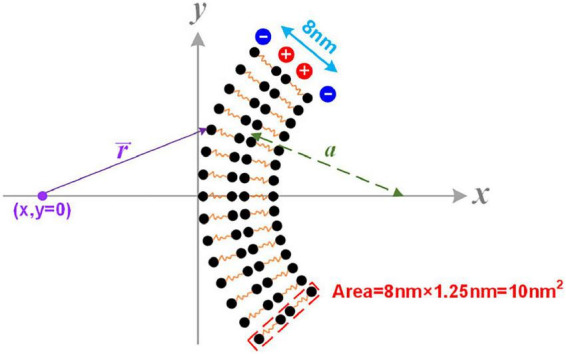
The modeling details of the calculation of the cell membrane’s dipole potential.

In Figure 4A, the axon membrane lipid bilayer consists of two layers of amphiphilic molecules. The positively charged hydrophobic tails of these lipids are directed toward the membrane center, while the negatively charged hydrophilic heads are directed toward the extra- and intracellular fluid (Monje, 2018). Each amphiphilic molecule is an electric dipole, a group of separated charges with opposite polarities. Thus, by arranging the position of each polar, the potential of a point as a function of the distance to the membrane can be calculated as shown in Figure 5. The arrangement of each polar is determined by the thickness of the bilayer and the diameter of the axon. Here axon radius, ***a***, is a variable. The total thickness of the lipid bilayer is 8 nm, a typical value of a cell membrane. The length of the dipole of each amphiphilic molecule is 3.6 nm, while the distance between the two positively charged polar is 0.8 nm. The cross-sectional area of each group of molecules is 10 nm^2^. The charge quantity of the n*_th_* polar is *q_n_*. The route from n*_th_* polar to the specific point (x, y = 0) on the *x*-axis is ${\vec{r}}_{n}$. The total electric potential at the position (x, y = 0) is the sum of the potential from each polar, as below:


φ=14⁢π⁢ε0⁢∑qn|rn⇀n|

Here *q_n_* is a value with a sign corresponding to the polarity of the charge. Considering the value of each *q_n_* is identical, so


φ=14⁢π⁢ε0⁢∑qn|rn⇀n|∝∑1|rn⇀n|

So


$$△\,V_{N\,1}={\varphi |}_{x=0\,n\,m}-{\varphi |}_{x=-8\,n\,m}$$

The potential, also called the dipole potential, generated by this lipid bilayer is shown in Figure 4A. Such dipole potential has two negative peaks at the extra- and intracellular surface and one positive peak at the membrane center (McLaughlin, 1989; Langner et al., 1990; McLaughlin and Murray, 2005; Leventis and Grinstein, 2010). The bending of the cell membrane will break the centrosymmetry of the bilayer structure and change the amplitude of those two negative peaks, called the flexoelectric effect (Petrov, 2002). In particular, the amplitude of the left negative peak located at the extracellular surface, named ***V**_*D*–*N1*_* (“D” refers to “dipole” and “N” refers to “negative”), decreases with bending, while the amplitude of the right negative peak located at the intracellular surface, named ***V**_*D*–*N2*_*, increases with bending. When the SC membrane contacts with the axon surface, a portion of ***V**_*D*–*N1*_*, labeled as Δ ***V**_*D*–*N1*_* in Figure 4B, is applied across SC’s membrane. This Δ ***V**_*D*–*N1*_* meets the criteria of growth promotion, which is an external negative E-field. Meanwhile, the amplitude of this Δ ***V**_*D*–*N1*_* increases with the axon caliber and saturates at a certain value (Figure 4C). Interestingly, in this modeling, Δ ***V**_*D*–*N1*_* has a sudden decline from a specific position at about 400 nm.

The surface potential of the cell membrane can influence the binding affinity of the peptide to lipid bilayers (Zhan and Lazaridis, 2012). So it is conjectured that the binding affinity between the polarized protein molecules on the SC membrane and axons, which are responsible for the interface adhesion, is positively correlated with the surface dipole potential of the axon Δ ***V**_*D*–*N1*_*. When the axon caliber is large, Δ ***V**_*D*–*N1*_* is strong enough for the molecules to form the bound; thus, SCs can grow and wrap on these axons to form myelin. However, when the axon caliber is lower than a certain value, e.g., 400 nm in Figure 4C, Δ ***V**_*D*–*N1*_* is insufficient to provide the binding affinity, leading to the failure of SC in adhering to the axon. It indicates that there is a threshold of axonal diameter to be myelinated, which is the observation of radial sorting of SCs. This threshold is about 1 μm is the actual observation. Considering that the modeling in this study is simplified and qualitative, it only indicates the existence of the threshold diameter rather than giving a precise value to it. Nevertheless, these simulation results suggest that the dipole potential from the axon surface can be one of the factors influencing myeline developments.

### An introspection of this model

In radial sorting, SCs can robustly identify the axons by their physical calibers. It means that the surface curvature is an important factor to be experienced by SCs. So it can be inferred that this physical identification signal is related to the surface curvature. The dipole potential is one of the candidates determined by the axon caliber and whose changing trend is consistent with Hypothesis-E. Since this Δ ***V**_*D*–*N1*_* is obtained from Hypothesis-E_*D*_, it is renamed as ***V***_*ED*_ to avoid confusion with other variables. Meanwhile, it is well-known that Nrg1 type III plays a crucial role in the myelination of SCs. A complete theory/model should account for this protein. We will have a detailed discussion in section “A more complete understanding of radial sorting.”

## Hypothesis-E_*P*_ to explain behaviors of Schwann cells

### Different behaviors of Schwann cells in myelination and remak bundle

The SCs behave differently in myelination and remak bundles (Feltri et al., 2016). In the scenario of myelination, an SC will wrap around a large axon with a 1:1 relationship. In the scenario of a remak bundle, an SC can never form myelination, even if a large axon is ensheathed.

### Hypothesis-E_*P*_

Hypothesis-E_*P*_ (P refers to “Positive”) is proposed to reveal the myelination criteria and explain the mechanism underlying differential SC activities:

*The growth of the inner tongue of myelin is inhibited by a positive E-field induced by action potentials. The strength of the E-field on the inner tongue is proportional to its capability of growth-inhibiting. When the E-field is lower than a threshold, it does not exert its inhibition function.*

**FIGURE 6:**
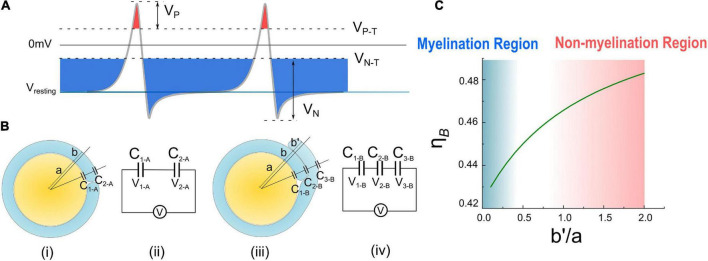
**(A)** The illustration of Hypothesis combining E_N_ and E_P_; **(B)** (i), (ii) the condition of case A with one layer of myelin and its equivalent circuit. (iii), (iv) The condition of case B with double- layer myelin and its equivalent circuit. **(C)** Calculate result of η *_*B*_* curve changes with $\frac{b^{\prime }}{a}$.

In Figure 6A, a new perspective about how myelin growth is modulated by E-field is shown. ***V***_*P*_ and ***V***_*N*_ refer to the amplitude of resting potential and the positive peak voltage of the action potential, respectively. The threshold voltage ***V**_*P*–*T*_* is the threshold voltage to inhibit myelin growth, while ***V**_*N*–*T*_* is the threshold voltage to promote myelin growth. The ratio between ***V**_*P*–*T*_* and ***V***_*P*_ is η *_*P*–*T*_*, and the ratio between ***V**_*N*–*T*_* and ***V***_*N*_ is η *_*N*–*T*_*. The area higher than ***V**_*P*–*T*_* is the inhibition phase (red area in Figure 6A), while the area lower than ***V**_*P*–*T*_* is the promotion phase of myelin growth (blue area in Figure 6A).

In Figures 6Bi,ii, the total voltage ***V*** (this voltage can be either the resting potential ***V***_*R*_ or the action potential ***V***_*A*_, “***A***” refers to “action”) across a single-layer myelin is applied on ***C_1–*A*_*** and ***C**_2–*A*_* (“A” refers to the capacitor of case A in Figure 6B):


$$C_{1-A}\propto a;$$

$$C_{2-A}\propto 2\times \left(a+b\right);$$

$$Q_{1-A}=C_{1-A}\times V_{1-A}=Q_{2-A}=C_{2-A}\times V_{2-A}=Q;$$

$$V_{1-A}+V_{2-A}=V;$$

Since ***C**_2–*A*_* only has a single layer of the cell membrane, the equivalent capacitance shall be doubled compared with the one with double layers of the cell membrane.

Then the ratio between the voltage on ***C**_1–*A*_* and ***V*** is:


$$\eta_{A}=\frac{V_{1-A}}{V}=\frac{1}{1+\frac{a}{2\,a+2\,b}}=\frac{1}{1+\frac{1}{2+2\times \frac{b}{a}}};$$

In Figures 6Biii,iv, the total voltage ***V*** across a double-layer myelin is applied on ***C**_1–*B*_*, ***C**_2–*B*_*, and ***C**_3–*B*_* (“B” refers to the capacitor of case B in Figure 6B):


$$C_{1-B}\propto a;$$

$$C_{2-B}\propto a+b;$$

$$C_{3-B}\propto 2\times \left(a+b+b^{\prime }\right);$$

$$Q_{1-B}=C_{1-B}\times V_{1-B}=Q_{2-B}=C_{2-B}\times V_{2-B}=Q_{3-B}=C_{3-B}\times V_{3-B}=Q;$$

$$V_{1}+V_{2}+V_{3}=V;$$

Here we set the thickness of the second layer is ***b***′, which is different from that of the first layer ***b***. Since ***C**_3–*B*_* only has a single layer of the cell membrane, its equivalent capacitance shall be doubled.

Then the ratio between the voltage on ***C**_1–*B*_* and ***V*** is:


$$\eta_{B}=\frac{V_{1-B}}{V}=\frac{1}{1+\frac{a}{a+b}+\frac{a}{2\,\left(a+b+b^{\prime }\right)}};$$

Since the myelin lamellae are not compact yet at the initial myelination process, ***b*** is a value comparable with ***a***. So here we set the ratio of ***b***/***a*** is 0.1, which is a typical value and a reasonable approximation, to further simplify the equation of η _*A*_ and η _*B*_ as below:


$$\eta_{A}=\frac{1}{1+\frac{1}{2+2\times \frac{b}{a}}}\approx 0.88;$$

$$\eta_{B}=\frac{1}{1+\frac{a}{a+b}+\frac{a}{2\,\left(a+b+b^{\prime }\right)}}=\frac{1}{1.909+\frac{1}{2\,\left(1.1+\frac{b^{\prime }}{a}\right)}};$$

As seen, η _*A*_ is a constant, meaning that about 88% of the transmembrane voltage, which can be either ***V***_*R*_ or ***V***_*A*_, will be applied onto the adaxonal layer of the myelin. Meanwhile, η **_*B*_** is a function of $\frac{b^{\prime }}{a}$, which is calculated as shown in Figure 6C. η *_*B*_* increases with $\frac{b^{\prime }}{a}$.

**FIGURE 7:**
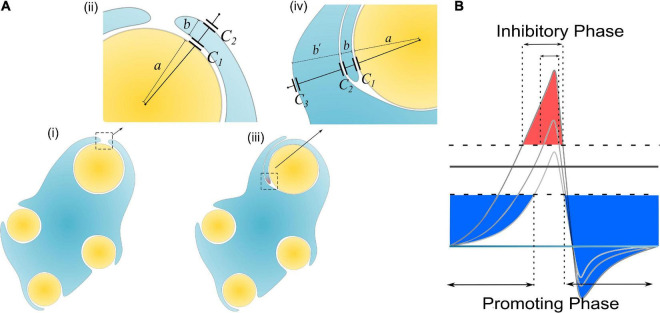
**(A)** The scenario when an SC of remak bundle ensheathes a large axon: (i, ii) Only one layer of SC is wrapped; (iii, iv) when the SC tries to wrap the second layer; **(B)** A more decayed action potential induces a shorter inhibitory phase (red region).

Then let’s consider the situations of the wrapping of the second myelin lamella on a large axon by both a normal SC and a remak SC, as shown in Figures 6B, 7A, respectively. For an SC forming myelination, the condition is similar to Figure 6B when *a*/*b*′. So its η_*B*_ is located within the blue region in Figure 6C, labeled with myelination region. For a remak SC, the condition is similar to Figure 6A. When a large axon is ensheathed by a remak bundle, initially the axon is wrapped by a SC as shown in Figures 7Ai,ii. When one of the SC terminal tends to further grow and wrap the large axon to form myelin, it inevitably faces the situation shown in Figures 7Aiii,iv when ***b***′ is comparative or even larger than ***a***. Thus its η *_*B*_* is located within a pink region in Figure 6C, labeled with the non-myelination region.

In Hypothesis-E_*P*_, a positive voltage ***V***_*P*_ in the action potential can inhibit myelin growth. Therefore, for a normal SC myelinating a large axon, the inhibiting voltage exerted upon the inner tongue is lower. The promoting factor induced by the negative voltage (mainly comes from the resting potential) dominates the myelin growth (see Figure 7B). However, for a remak SC, the inhibiting voltage upon the inner tongue is higher. Thus the inhibiting factor dominates the myelin growth, stopping the wrapping of the second layer. Since this inhibitory voltage on the inner tongue, ***V**_*P*_* × η *_*B*_*, is obtained from Hypothesis-E_*P*_, it is renamed as ***V***_*EP*_ to avoid confusion with other variables. This simulation under Hypothesis-E_*P*_ supports the experimental observation of the radial sorting (Monk et al., 2015), that is, (1) An SC can merely myelinate one axon. (2) Remak SC cannot myelinate. Moreover, we can also make a rough estimation of η *_*P*–*T*_*. It is a value located close to the myelination and non-myelination region interface 0.43∼0.46 shown in Figure 6C.

**FIGURE 8:**
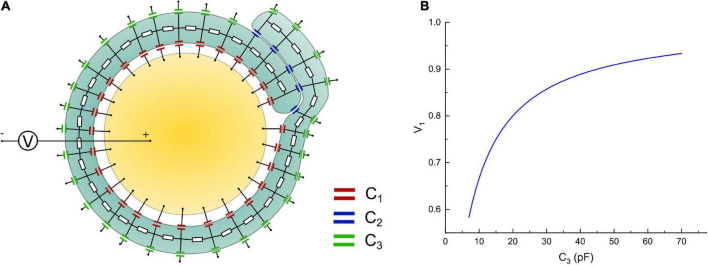
**(A)** A more detailed circuit model; **(B)** the voltage on the inner layer capacitor, C1, by increasing the capacitance of the outer layer, C3.

The modeling process shown in Figure 6 is an oversimplified model since the non-compact myelin sheath cannot be simplified as pure capacitors. We propose it just for consistency with the method in Figure 2. A More reasonable model with a circuit network by considering the cytoplasm in non-compact myelin sheath is shown in Figure 8A. The inner, middle, and outer layers are modeled as capacitors, respectively, C1, C2, and C3. The cytoplasmic fluid within the cell is modeled as resistors connecting the inner, middle, and outer layers. The modeling parameter is shown as follows:


$$C_{1}=5\,p\,F;$$

Considering that the middle layer consists of two layers of cell membrane, thus:


$$C_{2}=6pF||6pF=12pF;$$

Since we need to model the increasing of the outer layer, thus C3 should change within a range as follows:


$$C_{3}=7\,p\,F∼70\,p\,F;$$

The resistor modeling the cytoplasmic fluid is:


R=1⁢Ω;

Then we connect a voltage source, which models the action potential, with the intracellular and extracellular terminals. The voltage on one of the capacitors on the inner layer, which is C1, is measured by changing the value of C3. The simulation is performed in Simulink of MATLAB. The modeling results are shown in Figure 8B. As seen, the general trend of the voltage on the inner layer by increasing the outer layer is consistent with the result in Figure 6C. The exact numerical value is different. This is because by considering the conductive cytoplasmic channel connecting the inner and outer layers, the capacitors modeling the middle layer are short-circuited. Thus, all the voltage is shared only by the inner and outer layers, amplifying the voltage tuning effect by increasing the outer layer. Considering that all models here are for qualitative rather than quantitative study, we only focus on the general changing trend rather than the exact numerical value.

### Discussion

This model also indicates potential explanations for other experimental observations, as discussed below. Firstly, it is contradictory to the conventional understanding of the correlation between neural activities and myelin development. It was widely believed that the action potential is a positive factor in the myelination process (Monje, 2018), while in our model, it is a negative factor. If our model is correct, it can be predicted that by eliminating the action potential during myelin development, the myelin can grow thicker. Mayoral et al. (2018) have confirmed this hypermyelination phenomenon of oligodendrocytes by muting the action potential, which is supporting evidence of our model. It can be foreseen that the same phenomenon can be observed in the experiment of SCs. Secondly, the frequency of the action potential is also a factor affecting the fate of myelination. When the action potential is activated more frequently, which is the case of sensory fibers, the inhibiting factor tends to dominate, and the axons tend to be unmyelinated. Conversely, when the action potential is activated more rarely, which is the case of motor fibers, the promoting factor tends to dominate, and the axons tend to be myelinated. This may partially explain that the majority of the sensory fibers are unmyelinated while the counterparts of the motor fibers are myelinated (Schmalbruch, 1986). This model also indicates a positive correlation between neural hyperactivity and the degeneration of myelin. It may provide a clue for neurodegenerative disorders such as Parkinson’s disease, whose early stage symptoms, such as hand tremors and muscle stiffness, are the results of uncontrollable hyper-activation of some neurons, while the accompanying symptoms include the demyelination of neurons. At least, these phenomena are not contradictory to our model.

### A recap of the g-ratio phenomenon

**FIGURE 9:**
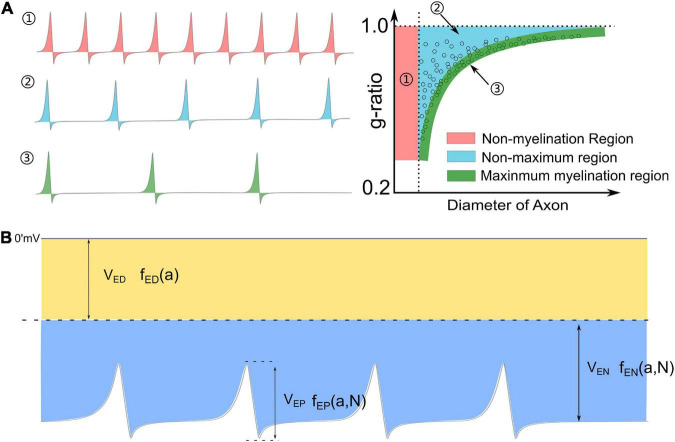
**(A)** The relationship between the frequency of action potential and the thickness of myelin: (1) An axon with a very high frequency of action potentials tends to be unmyelinated; (2) An axon with a medium frequency of action potentials cannot have maximum myelin thickness; (3) An axon with a very low frequency of action potentials tends to have maximum myelin thickness, forming the lower edge of the g-ratio data. **(B)** A complete perspective of Hypothesis-E: the total voltage consists of three major components: *V_*ED*_,V_*EN*_*, and ***V***_*EP*_.

The observation of the hypermyelination of oligodendrocytes by muting the action potential (Mayoral et al., 2018) reveals the relationship between myelination and neural activities. This observation indicates that an axon with fewer action potentials tends to have thicker myelin, while an axon with more action potentials tends to have thinner myelin. So the action potential is an inhibitory factor to the myelin growth, which agrees with our theory. Meanwhile, it also indicates a quantitative relationship between the frequency of action potential and the myelin thickness. An illustrative drawing of this quantitative relationship is shown in Figure 9A. For an axon with a very high frequency of action potential (Figure 9A1), it tends to be unmyelinated, which refers to the unmyelinated region (Region 1 in Figure 9A1). For an axon with a very low frequency of action potential, its myelin can grow to the maximum thickness, which refers to the lower edge (Region 3 in Figure 9A2). For the axon with a medium frequency of action potential, its myelin cannot grow to the maximum thickness, even at its mature state. This scenario refers to Region 2 in Figure 9A2. Then based on our theory, a complete explanation of the g-ratio phenomenon, including the non-myelination region, the lower edge, and the scattering data distribution, is proposed in Figure 9A. Moreover, our theory also makes another very interesting prediction. If unmyelinated axons tend to have a lower diameter and higher frequency of action potentials, it indicates a relationship between the action potential and the axonal diameter. In other words, the action potential is also an inhibitory factor to the radial growth of the axon.

### An introspection of this model

Currently, we cannot claim this is the exclusively correct model. But it is highly consistent with the whole theory. The behavior of SCs is determined by whether the inner tongue can further grow to form the second layer, which is still a growth issue. Since the inner tongue growth is affect by E-field in this theory, by leveraging the same circuit model and Hypothesis-E, we can easily acquire the explanatory model shown in Figure 7 without adding any new hypotheses.

## Discussion: A rethinking of the complete model

### The influence of the total voltage

At the beginning of this study, we have proposed the Hypothesis-E, which conjectures that the development of myelin is guided by an E-field applied upon the inner tongue. By explaining different phenomena of myelin development, it is concluded that this E-field consists of three components, as summarized below.

(1) The component, ***V***_*EN*_, from ***V***_*R*_. Although ***V***_*R*_ is almost an identical value for axons of different calibers, its component, ***V***_*EN*_, applied to the inner tongue changes with both the axon caliber and the number of myelin lamellae, explained in Figure 2. Therefore, ***V***_*EN*_ is a function of both the axon caliber, ***a***, and the number of myelin lamellae, ***N***:

$$V_{E\,N}=f_{E\,N}\,\left(a,N\right);$$

(2) The component, ***V***_*EP*_ from ***V***_*A*_. This ***V***_*EP*_ functions the same as ***V***_*EN*_ in the circuit, just with a different waveform. So it is also a function of ***a*** and ***N*** and changes with the same trend as ***V***_*EN*_:

$$V_{E\,P}=f_{E\,P}\,\left(a,N\right);$$

(3) The component, ***V***_*ED*_, is from the dipole potential of the cell membrane. This component does not change with the number of myelin lamellae, ***N***. So it is just a function of ***a***:

$$V_{E\,D}=f_{E\,D}\,\left(a\right);$$

The voltage upon the inner tongue, ***V***_*I*_, is the sum of these three components:


(8) $$V_{I}=f_{E\,N}\,\left(a,N\right)+f_{E\,P}\,\left(a,N\right)+f_{E\,D}\,\left(a\right);$$

The detailed waveform is shown in Figure 9B.

Since the major target of this study is to establish a new theoretical framework for the mechanism of myelin development, we do not intend to involve an accurately quantitative comparison of the importance of each component. However, a very rough and qualitative analysis can still help us have a better understanding. The amplitude of the dipole potential of the lipid membrane, whose measurement is not an easy task, is estimated within the range of 200∼1,000 mV (Brockman, 1994; Wang et al., 2006; Yang et al., 2008). It means ***V***_*ED*_, which is just a small portion of the dipole potential, as shown in Figure 4, may possess an amplitude of tens of mV, which is a comparative value to ***V***_*R*_ and ***V***_*A*_. Meanwhile, ***V***_*EN*_ and ***V***_*EP*_ take a small ratio of ***V***_*R*_ and ***V***_*A*_, respectively. Thus, ***V***_*ED*_ may take the major portion of ***V***_*I*_. In this scenario, ***V***_*I*_ has no substantial positive part. So a complete Hypothesis-E, which is a corrected version of Hypothesis-E_*P*_ in Figure 6A, is described below:

*The growth of the myelin is promoted by the negative E-field when it exceeds a threshold, represented by the potential **V**_*N1*–*T*_*, *and inhibited by the negative E-field when it is lowered than another threshold, represented by the potential **V**_*N2*–*T*_*, *respectively.*

Meanwhile, the conclusion about the g-ratio explanation in Figure 2 should also be corrected from two perspectives. Firstly, actual myelin growth is modulated by ***V***_*I*_, the sum of three components, rather than just one component assumed in Figure 2. Meanwhile, ***V***_*EN*_ and ***V***_*ED*_ have their own changing trends with the axonal diameter, and it is unclear how ***V***_*I*_ changes with axonal diameter. Therefore, the actual lower limit curve may deviate from the calculated one in Figure 3. The second correction comes from the different observations of myelin thickness. Some studies reported that the axon caliber is weakly correlated with the myelin thickness (Paus and Toro, 2009; FitzGibbon and Nestorovski, 2013; Stikov et al., 2015; Andersson et al., 2020). The number of myelin lamellae is normally lower than 50. However, we also notice that in some studies, it is reported that the myelin can have a perpetual growth, which makes the number of myelin lamellae more than 100 (Berthold et al., 1983; Fields, 2014). Meanwhile, in this scenario, a larger axon tends to have thicker myelin. It seems the divergence in Figure 3B can happen in some conditions. As explained in Figure 3C, it is because the voltage on the inner tongue (***V**_*ED*_+V_*EN*_*) is always higher than the threshold voltage. Considering that ***V***_*ED*_ does not decay with the increasing number of myelin lamellae, it is highly possible that ***V***_*ED*_ can solely provide the voltage to promote myelin growth. The myelin can grow perpetually with a constant growth rate, which agrees with the description in a previous study (Berthold et al., 1983), quoted here:

*It is, moreover, concluded that myelin production on the average seems to be a perpetual process which, in the fully mature cat, operates at the same rate regardless of axon size.*

**FIGURE 10:**
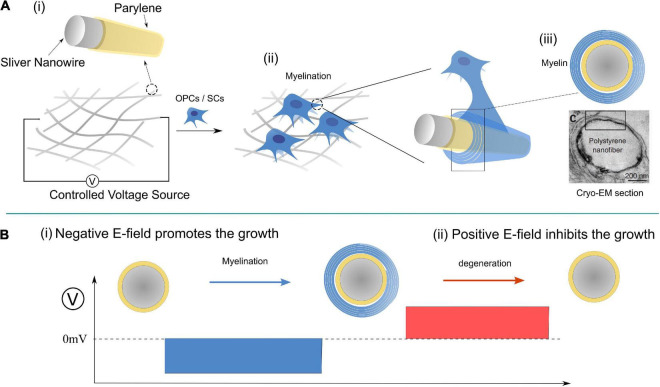
A designed experiment for validation of Hypothesis-E: **(A)** The experimental setup; **(B)** modulate the myelination process by controlling the E-field of the nano-wire.

A possible experiment for the validation of this theory is proposed in Figure 10, in which the applied E-field controls the myelination of non-axon fibers. It has been validated that axonal cues are not necessary for the myelin wrapping of oligodendrocytes, though they are still necessary for myelin compaction (Armati and Mathey, 2013). It is highly possible that SCs follow the same principle. So we can design an experiment shown in Figure 10 to validate the contribution of the E-field in myelin development. A mesh of silver micro/nano-wires, 0.2–10 μm in diameter, coated with 1 μm thick parylene as an insulating layer is used as a substitute for the axons with varying calibers. When it is partially immersed in the culture medium, the surface potential can be controlled by the applied voltage, as shown. The oligodendrocytes or SCs can both be cultured with nano-wire in the medium, and the myelination process can be observed by varying the applied voltage. Based on our theory, several phenomena can be predicted as follow:

(1) The minimum diameter of the myelinated wire decreases with the increasing amplitude of the negative voltage.
(2) When the positive voltage is applied, the myelination process will be inhibited for all wires.
(3) If a negative voltage is applied to induce the myelination first, the post-applied positive voltage can induce demyelination (Figure 10B).
(4) There will be a threshold voltage, ***VN1-T′***, to initiate the myelination process.
(5) There will be another threshold voltage, ***VN2-T′***, to initiate the demyelination.

### The paradox of neural-activity-dependent myelination

The paradox of this neural-activity-dependent (Foster et al., 2019) can be summarized as follow:

*Some studies observed that the action potential is a positive regulator for myelin growth, while in others, the action potential is a negative regulator.*

**FIGURE 11:**
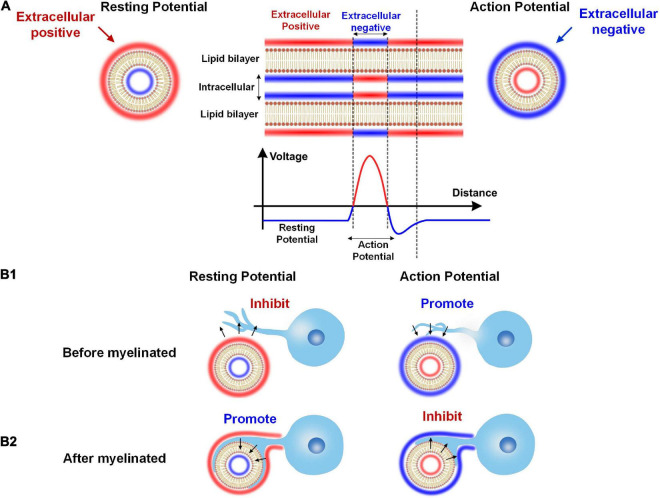
The dual effect of neural activity on myelination. **(A)** The extracellular E-field of neural activity on an axon: The resting potential induces a positive extracellular E-field, while the action potential induces a negative extracellular E-field. **(B1)** When the myelin cell has not attached to the axon, the positive extracellular E-field by the resting potential inhibits myelin growth, and the negative extracellular E-field by the action potential promotes myelin growth. **(B2)** When the myelin cell is attached to the axon, the resting and action potential effect will reserve.

This paradox can be easily solved by extending our theory, as shown in the Figure 11.

As shown in Figure 11A, the axon’s cell membrane is charged by E-field from either resting potential or action potential. As well-known, in the resting state, the extracellular is more positive than intracellular for an axon. So for resting potential, the extracellular is positive. Meanwhile, the action potential will flip the potential, making the extracellular negative. So, generally speaking, the extracellular is positive for the resting potential and is negative for the action potential.

This extracellular potential, or E-field, can modulate the myelin growth before the myelin cell (let’s say this is an oligodendrocyte) attach to the axon, as shown in Figure 11B1. Then according to our theory, the resting potential will inhibit the myelin attachment due to the positive E-field, while the action potential will promote the myelin attachment due to the negative E-field. So in this scenario, the action potential is a positive regulator for myelination.

However, the result will be the opposite once the myelin is attached to the axon, as shown in Figure 11B2. This is because once the myelin cell contacts the axon, the direction of the E-field will be opposite. The cell membrane of the myelin cell attached to the axon can experience the intracellular E-field. So for the resting potential, the intracellular negative E-field will promote the inner tongue growth. But for the action potential, the intracellular positive E-field will inhibit the inner tongue growth. So in this scenario, the action potential is a negative regulator for myelination.

According to the above explanation, we can conclude that:

(1) The action potential is a promoting factor for forming new myelin sheaths. So if we only investigate the number of myelin sheaths, the action potential is a positive regulator. This is the phenomenon observed by a majority group of neural-activity-dependent myelination (Fields, 2015). The rat was trained for several hours each day, and the motor cortex will have more myelin (Kleim et al., 2002; Sampaio-Baptista et al., 2013). In this study, both action and resting potential exert their functions on myelination. During the training, the action potential can initiate the new myelination wrapping. But the training only lasts several hours each day. It means that for the rest 20 h each day, the resting potential increases the thickness of the myelin sheaths. So in this study, both the increment of new myelin and sheath thickening can be observed. However, people did not recognize the function of resting potential to myelin growth. Therefore, the initiation of myelin wrapping and the further myelin thickening are all attributed to the role of action potentials.
(2) The action potential is an inhibiting factor for inner tongue growth after the myelin sheath is formed. So if we only investigate the thickness of a formed myelin sheath, the action potential is a negative regulator. Jonah Chan’s group observed this phenomenon in the experiment on optic fibers (Mayoral et al., 2018). In Jonah Chan’s study, the action potentials of the optic fibers are entirely removed. As a result, the myelin sheaths grow thicker, called hypermyelination. This experiment perfectly shows the promoting effect of resting potential on myelin growth. Meanwhile, it also indicates that the action potential is an inhibitory factor to myelin growth.

### A revision of the g-ratio model

The fitting curves in Figure 3E are manually plotted to indicate the existence of the lower limit. They are not based on specific modeling parameters. An accurate data fitting is impossible according to the g-ratio model in section “Modeling the relationship between g-ratio and the E-field.” The primary reason is that the model is not complete in section “Modeling the relationship between g-ratio and the E-field.” As we mentioned in section “The influence of the total voltage,” a more comprehensive model should also consider the voltage ***V***_*ED*_ from dipole potential.

Now three voltage components need to be considered in the g-ratio calculation:

(1) ***V***_*EN*_ is the voltage component from the resting potential. Since this component decays with the increasing lamellae layers, the growth of the inner tongue tends to terminate at a particular layer. So ***V***_*EN*_ is the factor that determines the myelin sheath will have a maximum layer, meaning that the g-ratio shall have a lower limit.
(2) ***V***_*ED*_ is the component from the dipole potential. This component does not decay with the increasing lamellae layers. Since it is also part of the total voltage to induce the myelin growth, this ***V***_*ED*_ will change the actual shape of the lower limit of the g-ratio.
(3) ***V***_*T*_ is the threshold voltage required for myelin growth. Section “Modeling the relationship between g-ratio and the E-field” considers this voltage as a ratio, η *_*N*–*T*_*, to the resting potential, which is a constant. But now, we need to add the dipole potential, a function of axonal diameter. Therefore, this ***V***_*T*_ cannot be simplified as a ratio but be considered as an actual voltage.

When all these three parameters, ***V***_*EN*_, ***V**_*ED*_*, and ***V***_*T*_, are given, a g-ratio curve can be calculated. ***V***_*EN*_ is a parameter that a given number of layers can calculate. ***V***_*ED*_ is a parameter whose actual value is unknown. We only know its general changing trend shown in Figure 4C. ***V***_*T*_ is also an unkown parameter. Therefore, we cannot generate all the possible g-ratio just by changing ***V***_*T*_.

**FIGURE 12:**
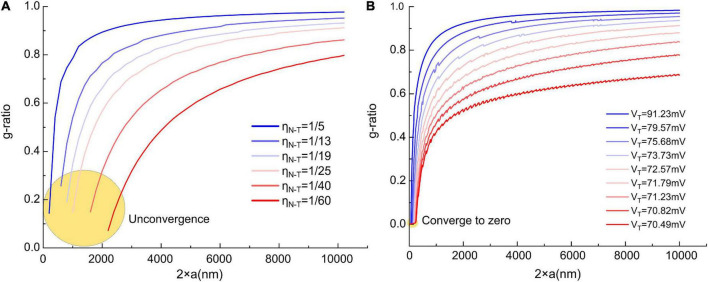
The comparison of the g-ratio curves before and after the correction. **(A)** The g-ratio curves before the correction, these curves are not converged to zero point; **(B)** the g-ratio curves after the correction, all curves are converged to zero point.

But we still can give a proper ***V***_*ED*_ and demonstrate a more corrected g-ratio modeling result. We rescale the curve in Figure 4C to the same amplitude of the resting potential, which is 70 mV, which means the maximum value of the dipole potential is 70 mV. So now, ***V***_*ED*_ is a known parameter. Then we change ***V***_*T*_ from 70.49 to 91.23 mV to generate the g-ratio curve. A comparison of the g-ratio modeling results before and after the correction is shown in Figure 12. Before the correction (Figure 12A), those g-ratio curves do not converge to the origin point. But after the correction, all g-ratio curves converge to the origin point (Figure 12B). According to a detailed observation of those g-ratio data in Figure 3E, the lower limit curves converge to the origin point, which agrees with our correction.

**FIGURE 13:**
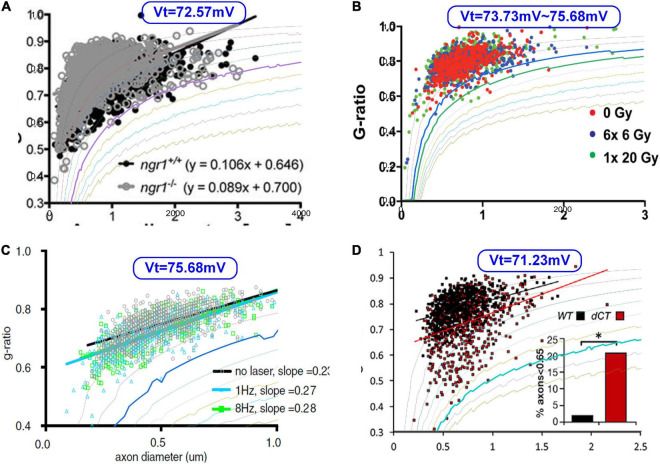
The fitting of the lower limits of g-ratio data. **(A)** Lee et al. (2017); **(B)** Begolly et al. (2016); **(C)** Piscopo et al. (2018); **(D)** Elazar et al. (2019).

Then we try fitting some g-ratio data with our corrected g-ratio curve, as shown in Figure 13. Since we do not have the original data published in other studies (Begolly et al., 2016; Lee et al., 2017; Piscopo et al., 2018; Elazar et al., 2019), we can only overlap our modeling curves with the data figures to show how good the fitting is. The most fitting curves are plotted as thicker lines. The corresponding ***V***_*T*_ is also shown in each figure. These four cases show that our modeling results can better fit the lower limit.

Here we need to emphasize several points.

(1) Our modeling results show that by adding ***V***_*ED*_, the calculated curves are closer to the experimental results. So this correction is quite necessary.
(2) A good fitting does not necessarily mean our modeling parameter is correct. Since there are infinite combinations of ***V***_*ED*_ and ***V***_*T*_, we cannot know the actual value of these two parameters just by modeling. In other studies, there are also many experimental data that the parameters in Figure 3B cannot fit. The deviation mainly comes from the slope and curvature, which can be tuned by both ***V***_*ED*_ and ***V***_*T*_.
(3) Our theory provides a framework to understand these phenomena rather than predicting the actual parameters. Therefore, pursuing the modeling accuracy at the current stage is meaningless and impossible.

Now we also can explain the unknown factor mentioned in section “The relation between the divergence region and unmyelinated axons” that can eliminate the divergence in Figure 3B. As shown in Figure 12B, we correct the modeling of the lower edge of g-ratio by considering the influence of the dipole potential. The major difference is that all curves are converged to zero point, which means the lower limit of g-ratio is zero only when the axonal diameter is also zero. The divergence in Figure 3B means that the curve of the g-ratio’s lower edge goes to zero. Before the correction, which is shown in Figure 12A, the curves go to zero when the axonal diameter is a non-zero value. This is the reason for the divergence region. However, after the correction in Figure 12B, all curves reach zero only when the axonal diameter is a zero value. An axon with a diameter of zero is biologically impossible. Thus the divergence is eliminated.

### A more complete understanding of radial sorting

It is known that the Nrg1 type III plays a critical role in myelination (Nave and Salzer, 2006). However, Nrg1 type III is not the sole factor for myelination. It is confirmed that the CHO cell with Nrg1 expression cannot be myelinated by SCs (Taveggia et al., 2005). So apart from the Nrg1, there is still a missing factor to control the fate of SCs. In our theory, this factor is E-field, the sum of voltages from resting, action, and dipole potentials. It is known that for oligodendrocytes, Nrg1 is not required. Thus our theory can perfectly be applied. However, Nrg1 is necessary for SCs. So it can be inferred that there is a protein that can sense the E-field to control the growth of SCs, on the SC surface. The E-field sensing function is activated when it is bonded with the Nrg1 on the axonal surface. This is the reason that CHO cells with Nrg1 expression, which has a much lower resting potential (∼-10 mv) and no action potential, cannot be myelinated. So generally, our theory still remains the same. But for SCs, the theory should be extended to include the effect of Nrg1.

## Conclusion

Our simulation suggests that myelin development can be modulated by E-field. This E-field is induced by three origins: the resting potential, the action potential, and the dipole potential. Each has its unique changing patterns with the axonal caliber and the number of myelin lamellae. Our model can be used to explain a series of observed phenomena during myelin development, such as radial sorting and g-ratio. Furthermore, our model reveals that the myelination process can be controlled by physical factors, bridging neural electrical activities and neural development.

## Data availability statement

The original contributions presented in this study are included in the article, further inquiries can be directed to the corresponding author/s.

## Author contributions

HW proposed the theory. YL carried out the modeling process. TG helped refine the theory and improve the writing. WY, TZ, YZ, RZ, BS, FL, YH, and TW contributed to the reference collection, idea discussion, and early state of the theory establishment. SY helped plot figures and search for the references for manuscript revision. All authors contributed to the article and approved the submitted version.

## References

[B1] Andersson M. Kjer H. M. Rafael-Patino J. Pacureanu A. Pakkenberg B. Thiran J. P. (2020). Axon morphology is modulated by the local environment and impacts the noninvasive investigation of its structure–function relationship. *Proc. Natl. Acad. Sci. U.S.A.* 117 33649–33659. 10.1073/pnas.2012533117 33376224 PMC7777205

[B2] Armati P. J. Mathey E. K. (2013). An update on Schwann cell biology—immunomodulation, neural regulation and other surprises. *J. Neurol. Sci.* 333 68–72. 10.1016/j.jns.2013.01.018 23422027

[B3] Barbizan R. Castro M. V. Ferreira R. S. Barraviera B. Oliveira A. L. (2014). Long-term spinal ventral root reimplantation, but not bone marrow mononuclear cell treatment, positively influences ultrastructural synapse recovery and motor axonal regrowth. *Int. J. Mol. Sci.* 15 19535–19551. 10.3390/ijms151119535 25353176 PMC4264127

[B4] Begolly S. Shrager P. G. Olschowka J. A. Williams J. P. O’Banion M. K. (2016). Fractionation spares mice from radiation-induced. *Int. J. Radiat. Oncol. Biol. Phys.* 96 449–457. 10.1016/j.ijrobp.2016.05.005 27478169 PMC5014650

[B5] Bercury K. K. Dai J. Sachs H. H. Ahrendsen J. T. Wood T. L. Macklin W. B. (2014). Conditional ablation of raptor or rictor has differential impact on oligodendrocyte differentiation and CNS myelination. *J. Neurosci.* 34 4466–4480. 10.1523/JNEUROSCI.4314-13.2014 24671993 PMC3965777

[B6] Berthold C. H. Nilsson I. Rydmark M. (1983). Axon diameter and myelin sheath thickness in nerve fibres of the ventral spinal root of the seventh lumbar nerve of the adult and developing cat. *J. Anat.* 136(Pt 3) 483–508. 6885614 PMC1171896

[B7] Brockman H. (1994). Dipole potential of lipid membranes. *Chem. Phys. Lipids* 73 57–79. 10.1016/0009-3084(94)90174-0 8001185

[B8] Bunge R. P. Bunge M. B. Bates M. (1989). Movements of the Schwann cell nucleus implicate progression of the inner (axon-related) Schwann cell process during myelination. *J. Cell Biol.* 109 273–284. 10.1083/jcb.109.1.273 2745552 PMC2115485

[B9] Chomiak T. Hu B. (2009). What is the optimal value of the g-ratio for myelinated fibers in the rat CNS? A theoretical approach. *PLoS One* 4:e7754. 10.1371/journal.pone.0007754 19915661 PMC2771903

[B10] Colognato H. Franklin R. J. (2004). The mysteries of myelin unwrapped. *Science* 304 688–689. 10.1126/science.1097851 15118149

[B11] Dimas P. Montani L. Pereira J. A. Moreno D. Trötzmüller M. Gerber J. (2019). CNS myelination and remyelination depend on fatty acid synthesis by oligodendrocytes. *Elife* 8:e44702. 10.7554/eLife.44702.030 31063129 PMC6504237

[B12] Dutta D. J. Woo D. H. Lee P. R. Pajevic S. Bukalo O. Huffman W. C. (2018). Regulation of myelin structure and conduction velocity by perinodal astrocytes. *Proc. Natl. Acad. Sci. U.S.A.* 115 11832–11837. 10.1073/pnas.1811013115 30373833 PMC6243273

[B13] Elazar N. Vainshtein A. Golan N. Vijayaragavan B. Schaeren-Wiemers N. Eshed-Eisenbach Y. (2019). Axoglial adhesion by Cadm4 regulates CNS myelination. *Neuron* 101 224–231. 10.1016/j.neuron.2018.11.032 30551998 PMC6371057

[B14] Fanarraga M. L. Griffiths I. R. Zhao M. Duncan I. D. (1998). Oligodendrocytes are not inherently programmed to myelinate a specific size of axon. *J. Comp. Neurol.* 399 94–100. 10.1002/(SICI)1096-9861(19980914)399:1<94::AID-CNE7>3.0.CO;2-5 9725703

[B15] Feltri M. L. Poitelon Y. Previtali S. C. (2016). How Schwann cells sort axons: New concepts. *Neuroscientist* 22 252–265. 10.1177/1073858415572361 25686621 PMC5181106

[B16] Ferreira S. Pitman K. A. Summers B. S. Wang S. Young K. M. Cullen C. L. (2021). Oligodendrogenesis increases in hippocampal grey and white matter prior to locomotor or memory impairment in an adult mouse model of tauopathy. *Eur. J. Neurosci.* 54 5762–5784. 10.1111/ejn.14726 32181929 PMC8451881

[B17] Fields R. D. (2014). Myelin formation and remodeling. *Cell* 156 15–17. 10.1016/j.cell.2013.12.038 24439366 PMC5017146

[B18] Fields R. D. (2015). A new mechanism of nervous system plasticity: Activity-dependent myelination. *Nat. Rev. Neurosci.* 16 756–767. 10.1038/nrn4023 26585800 PMC6310485

[B19] Fields R. D. Bukalo O. (2020). Myelin makes memories. *Nat. Neurosci.* 23 469–470. 10.1038/s41593-020-0606-x 32094969 PMC8240098

[B20] FitzGibbon T. Nestorovski Z. (2013). Human intraretinal myelination: Axon diameters and axon/myelin thickness ratios. *Indian J. Ophthalmol.* 61:567. 10.4103/0301-4738.121075 24212308 PMC3853453

[B21] Foster A. Y. Bujalka H. Emery B. (2019). Axoglial interactions in myelin plasticity: Evaluating the relationship between neuronal activity and oligodendrocyte dynamics. *Glia* 67 2038–2049. 10.1002/glia.23629 31038804

[B22] Fraher J. P. (1972). A quantitative study of anterior root fibres during early myelination. *J. Anat.* 112(Pt 1) 99–124. 5086215 PMC1271346

[B23] Ghidinelli M. Poitelon Y. Shin Y. K. Ameroso D. Williamson C. Ferri C. (2017). Laminin 211 inhibits protein kinase a in Schwann cells to modulate neuregulin 1 type III-driven myelination. *PLoS Biol.* 15:e2001408. 10.1371/journal.pbio.2001408 28636612 PMC5479503

[B24] Griffin J. W. Thompson W. J. (2008). Biology and pathology of nonmyelinating Schwann cells. *Glia* 56 1518–1531. 10.1002/glia.20778 18803315

[B25] Harty B. L. Monk K. R. (2017). Unwrapping the unappreciated: Recent progress in Remak Schwann cell biology. *Curr. Opin. Neurobiol.* 47 131–137. 10.1016/j.conb.2017.10.003 29096241 PMC5963510

[B26] Harty B. L. Coelho F. Pease-Raissi S. E. Mogha A. Ackerman S. D. Herbert A. L. (2019). Myelinating Schwann cells ensheath multiple axons in the absence of E3 ligase component Fbxw7. *Nat. Commun.* 10 1–12. 10.1038/s41467-019-10881-y 31278268 PMC6611888

[B27] Hildebrand C. (1971). Ultrastructural and light-microscopic studies of the nodal region in large myelinated fibres of the adult feline spinal cord white matter. *Acta Physiol. Scand.* 364 43–79. 10.1111/j.1365-201X.1971.tb10978.x 4109394

[B28] Hines J. H. Ravanelli A. M. Schwindt R. Scott E. K. Appel B. (2015). Neuronal activity biases axon selection for myelination in vivo. *Nat. Neurosci.* 18 683–689. 10.1038/nn.3992 25849987 PMC4414883

[B29] Höke A. Ho T. Crawford T. O. LeBel C. Hilt D. Griffin J. W. (2003). Glial cell line-derived neurotrophic factor alters axon schwann cell units and promotes myelination in unmyelinated nerve fibers. *J. Neurosci.* 23 561–567. 10.1523/JNEUROSCI.23-02-00561.2003 12533616 PMC6741898

[B30] Ikeda M. Oka Y. (2012). The relationship between nerve conduction velocity and fiber morphology during peripheral nerve regeneration. *Brain Behav.* 2 382–390. 10.1002/brb3.61 22950042 PMC3432961

[B31] Kleim J. A. Barbay S. Cooper N. R. Hogg T. M. Reidel C. N. Remple M. S. (2002). Motor learning-dependent synaptogenesis is localized to functionally reorganized motor cortex. *Neurobiol. Learn. Mem.* 77 63–77. 10.1006/nlme.2000.4004 11749086

[B32] Klok M. D. Bugiani M. de Vries S. I. Gerritsen W. Breur M. van der Sluis S. (2018). Axonal abnormalities in vanishing white matter. *Ann. Clin. Transl. Neurol.* 5 429–444. 10.1002/acn3.540 29687020 PMC5899913

[B33] Langner M. Cafiso D. Marcelja S. McLaughlin S. (1990). Electrostatics of phosphoinositide bilayer membranes. Theoretical and experimental results. *Biophys. J.* 57 335–349. 10.1016/S0006-3495(90)82535-2 2156577 PMC1280674

[B34] Lee J. Y. Kim M. J. Li L. Velumian A. A. Aui P. M. Fehlings M. G. (2017). Nogo receptor 1 regulates Caspr distribution at axo-glial units in the central nervous system. *Sci. Rep.* 7:8958. 10.1038/s41598-017-09405-9 28827698 PMC5567129

[B35] Lemke G. (1988). Unwrapping the genes of myelin. *Neuron* 1 535–543. 10.1016/0896-6273(88)90103-1 2483101

[B36] Leventis P. A. Grinstein S. (2010). The distribution and function of phosphatidylserine in cellular membranes. *Ann. Rev. Biophys.* 39 407–427. 10.1146/annurev.biophys.093008.131234 20192774

[B37] Liu Y. Zhang Y. Yue W. Zhu R. Guo T. Liu F. (2021a). A physical answer to Peters quadrant mystery: A modeling study. *arXiv* [Preprint]. arXiv:2111.11690

[B38] Liu Y. Zhang Y. Yue W. Zhu R. Guo T. Liu F. (2021b). A Physical perspective to understand the mechanism of myelin development. *arXiv* [Preprint]. arXiv:2111.13689

[B39] Marro B. S. Skinner D. D. Cheng Y. Grist J. J. Dickey L. L. Eckman E. (2019). Disrupted CXCR2 signaling in oligodendroglia lineage cells enhances myelin repair in a viral model of multiple sclerosis. *J. Virol.* 93 e240–e219. 10.1128/JVI.00240-19 31243125 PMC6714798

[B40] Mayoral S. R. Etxeberria A. Shen Y. A. A. Chan J. R. (2018). Initiation of CNS myelination in the optic nerve is dependent on axon caliber. *Cell Rep.* 25 544–550. 10.1016/j.celrep.2018.09.052 30332636 PMC6258034

[B41] McLaughlin S. (1989). The electrostatic properties of membranes. *Ann. Rev. Biophys. Biophys. Chem.* 18 113–136. 10.1146/annurev.bb.18.060189.000553 2660821

[B42] McLaughlin S. Murray D. (2005). Plasma membrane phosphoinositide organization by protein electrostatics. *Nature* 438 605–611. 10.1038/nature04398 16319880

[B43] Michailov G. V. Sereda M. W. Brinkmann B. G. Fischer T. M. Haug B. Birchmeier C. (2004). Axonal neuregulin-1 regulates myelin sheath thickness. *Science* 304 700–703. 10.1126/science.1095862 15044753

[B44] Monje M. (2018). Myelin plasticity and nervous system function. *Ann. Rev. Neurosci.* 41 61–76. 10.1146/annurev-neuro-080317-061853 29986163

[B45] Monk K. R. Feltri M. L. Taveggia C. (2015). New insights on Schwann cell development. *Glia* 63 1376–1393. 10.1002/glia.22852 25921593 PMC4470834

[B46] Nave K. A. Salzer J. L. (2006). Axonal regulation of myelination by neuregulin 1. *Curr. Opin. Neurobiol.* 16 492–500. 10.1016/j.conb.2006.08.008 16962312

[B47] Nave K. A. Werner H. B. (2014). Myelination of the nervous system: Mechanisms and functions. *Ann. Rev. Cell Dev. Biol.* 30 503–533. 10.1146/annurev-cellbio-100913-013101 25288117

[B48] Ommer A. Figlia G. Pereira J. A. Datwyler A. L. Gerber J. DeGeer J. (2019). Ral GTPases in Schwann cells promote radial axonal sorting in the peripheral nervous system. *J. Cell Biol.* 218 2350–2369. 10.1083/jcb.201811150 31201267 PMC6605813

[B49] Orita S. Henry K. Mantuano E. Yamauchi K. De Corato A. Ishikawa T. (2013). Schwann cell LRP1 regulates remak bundle ultrastructure and axonal interactions to prevent neuropathic pain. *J. Neurosci.* 33 5590–5602. 10.1523/JNEUROSCI.3342-12.2013 23536074 PMC3837698

[B50] Paus T. Toro R. (2009). Could sex differences in white matter be explained by g ratio? *Front. Neuroanat.* 3:14. 10.3389/neuro.05.014.2009 19753325 PMC2742663

[B51] Peters A. (1961). A radial component of central myelin sheaths. *J. Cell Biol.* 11 733–735. 10.1083/jcb.11.3.733 14485724 PMC2225133

[B52] Peters A. (1964). Further observations on the structure of myelin sheaths in the central nervous system. *J. Cell Biol.* 20 281–296. 10.1083/jcb.20.2.281 14126873 PMC2106395

[B53] Petrov A. G. (2002). Flexoelectricity of model and living membranes. *Biochim. Biophys. Acta* 1561 1–25. 10.1016/S0304-4157(01)00007-7 11988178

[B54] Piscopo D. M. Weible A. P. Rothbart M. K. Posner M. I. Niell C. M. (2018). Changes in white matter in mice resulting from low-frequency brain stimulation. *Proc. Natl. Acad. Sci. U.S.A.* 115 E6339–E6346. 10.1073/pnas.1802160115 29915074 PMC6142236

[B55] Richards W. Kalil R. Moore C. L. (1983). An observation about myelination. *Exp. Brain Res.* 52 219–225. 10.1007/BF00236630 6641884

[B56] Ronchi G. Jager S. B. Vaegter C. B. Raimondo S. Giacobini-Robecchi M. G. Geuna S. (2014). Discrepancies in quantitative assessment of normal and regenerated peripheral nerve fibers between light and electron microscopy. *J. Peripher. Nerv. Syst.* 19 224–233. 10.1111/jns.12090 25418762

[B57] Sampaio-Baptista C. Khrapitchev A. A. Foxley S. Schlagheck T. Scholz J. Jbabdi S. (2013). Motor skill learning induces changes in white matter microstructure and myelination. *J. Neurosci.* 33 19499–19503. 10.1523/JNEUROSCI.3048-13.2013 24336716 PMC3858622

[B58] Sanders F. K. Whitteridge D. (1946). Conduction velocity and myelin thickness in regenerating nerve fibres. *J. Physiol.* 105 152–174. 10.1113/jphysiol.1946.sp004160 20999939

[B59] Schmalbruch H. (1986). Fiber composition of the rat sciatic nerve. *Anat. Rec.* 215 71–81. 10.1002/ar.1092150111 3706794

[B60] Stadelmann C. Timmler S. Barrantes-Freer A. Simons M. (2019). Myelin in the central nervous system: Structure, function, and pathology. *Physiol. Rev.* 99 1381–1431. 10.1152/physrev.00031.2018 31066630

[B61] Stikov N. Campbell J. S. Stroh T. Lavelée M. Frey S. Novek J. (2015). Quantitative analysis of the myelin g-ratio from electron microscopy images of the macaque corpus callosum. *Data Brief* 4 368–373. 10.1016/j.dib.2015.05.019 26217818 PMC4510539

[B62] Taveggia C. Zanazzi G. Petrylak A. Yano H. Rosenbluth J. Einheber S. (2005). Neuregulin-1 type III determines the ensheathment fate of axons. *Neuron* 47 681–694. 10.1016/j.neuron.2005.08.017 16129398 PMC2387056

[B63] Uzman B. G. Nogueira-Graf G. (1957). Electron microscope studies of the formation of nodes of Ranvier in mouse sciatic nerves. *J. Cell Biol.* 3 589–598. 10.1083/jcb.3.4.589 13449102 PMC2224104

[B64] Wang H. Wang J. Cai G. Liu Y. Qu Y. Wu T. (2021). A physical perspective to the inductive function of myelin—a missing piece of neuroscience. *Front. Neural Circuits* 14:562005. 10.3389/fncir.2020.562005 33536878 PMC7848263

[B65] Wang L. Bose P. S. Sigworth F. J. (2006). Using cryo-EM to measure the dipole potential of a lipid membrane. *Proc. Natl. Acad. Sci. U.S.A.* 103 18528–18533. 10.1073/pnas.0608714103 17116859 PMC1693696

[B66] Webster H. D. (1971). The geometry of peripheral myelin sheaths during their formation and growth in rat sciatic nerves. *J. Cell Biol.* 48 348–367. 10.1083/jcb.48.2.348 4928020 PMC2108190

[B67] Webster H. D. Martin J. R. O’Connell M. F. (1973). The relationships between interphase Schwann cells and axons before myelination: A quantitative electron microscopic study. *Dev. Biol.* 32 401–416. 10.1016/0012-1606(73)90250-9 4789698

[B68] Xie F. Liang P. Fu H. Zhang J. C. Chen J. (2014). Effects of normal aging on myelin sheath ultrastructures in the somatic sensorimotor system of rats. *Mol. Med. Rep.* 10 459–466. 10.3892/mmr.2014.2228 24818843

[B69] Yang Y. Mayer K. M. Wickremasinghe N. S. Hafner J. H. (2008). Probing the lipid membrane dipole potential by atomic force microscopy. *Biophys. J.* 95 5193–5199. 10.1529/biophysj.108.136507 18805919 PMC2586574

[B70] Zhan H. Lazaridis T. (2012). Influence of the membrane dipole potential on peptide binding to lipid bilayers. *Biophys. Chem.* 161 1–7. 10.1016/j.bpc.2011.10.002 22100997 PMC3262865

[B71] Zhao J. X. Ohnishi A. Itakura C. Mizutani M. Yamamoto T. Hojo T. (1995). Smaller axon and unaltered numbers of microtubules per axon in relation to number of myelin lamellae of myelinated fibers in the mutant quail deficient in neurofilaments. *Acta Neuropathol.* 89 305–312. 10.1007/BF00309623 7610761

[B72] Zheng H. Chang L. Patel N. Yang J. Lowe L. Burns D. K. (2008). Induction of abnormal proliferation by nonmyelinating schwann cells triggers neurofibroma formation. *Cancer Cell* 13 117–128. 10.1016/j.ccr.2008.01.002 18242512

[B73] Zuckerkandl E. Pauling L. (1965). “Evolutionary divergence and convergence in proteins”, in *Evolving genes and proteins*, eds Vernon B. Henry J. V. (Cambridge: Academic Press), 97–166. 10.1016/B978-1-4832-2734-4.50017-6

